# Equitable Representation of Race, Ethnicity, and Ancestry Among Genomic Studies of Preterm Birth: A Systematic Review

**DOI:** 10.7759/cureus.53757

**Published:** 2024-02-07

**Authors:** Liberty Reforma, Simone Greenberg, Rachel Ledyard, Heather Burris

**Affiliations:** 1 Obstetrics and Gynecology, Boston Medical Center, Boston, USA; 2 School of Public Health, Columbia University College of Physicians and Surgeons, New York, USA; 3 Biostatistics, Children's Hospital of Philadelphia, Philadelphia, USA; 4 Neonatology, Children's Hospital of Philadelphia, Philadelphia, USA

**Keywords:** birth, preterm, genome-wide association studies, genetics, race, equity, preterm birth, genomics

## Abstract

We conducted a systematic review of representation of race, ethnicity, and ancestry among genomic studies of preterm birth. Our data sources included CINHAL, EMBASE, MEDLINE (PubMed), and Scopus. Studies were included if they were human, genomic studies of preterm birth that analyzed greater than 1,000 genes and included race, ethnicity, and/or ancestry information. Two authors independently reviewed all abstracts and full-text manuscripts. Twelve studies were included. Ancestry was reported for 139,189 (93.6%) participants. Race was reported for 4,841 (3.3%) participants and ethnicity was reported for 7,154 (5.0%) participants. Of the 148,644 births represented in this systematic review, over 90% were reported to be of European ancestry, and race and ethnicity were not further described. When examining the smaller subset of individuals described by race alone, 2,444 individuals were identified as Black or African American and 1,853 were identified as White. Race, ethnicity, and ancestry were not reported in a uniform manner, which makes ascertainment of the genetic contribution to population differences in preterm birth inequities impossible. When reported as race, ethnicity and ancestry, Black or African American populations were under-represented among the studies in this review. Research of the genomics of preterm birth not only requires increased representation of populations that are disproportionately affected, but it also requires standardized reporting of race, ethnicity, and ancestry.

## Introduction and background

Preterm birth defined as birth prior to 37 completed weeks of gestation, is one of the largest contributors to perinatal morbidity and mortality worldwide. Every year, an estimated 15 million infants are born preterm [1] and yet the etiology of preterm birth remains poorly understood. Longstanding racial inequities in preterm birth are well-described; in the US, Black individuals are 50% more likely to have preterm birth than White individuals [2]. Little progress to reduce inequity has been made, with racial inequities in preterm birth persisting for decades.

Race is a social construct used to group and classify people, but historically has led to the marginalization of some groups [3]. As described by the National Human Genome Research Institute, race has been used to establish a hierarchy, leading individuals to be treated differently, resulting in racism [4]. Black, Hispanic, and indigenous groups in the US are disproportionately affected by adverse health conditions but are under-represented in clinical trials [5-7]. Despite the conceptualization of race as a social construct, researchers have searched for genetic reasons for disparities in preterm birth [8-9]. However, before any determination of a genetic basis for disparities in preterm birth could be made, genetics would first need to be shown to contribute to preterm birth generally. Second, there would need to be adequate representation of Black and White individuals in genomic studies of preterm birth. While studies have shown subtle associations of specific genes with preterm birth, there is not a definitive genotype identified that causes preterm birth [10-11]. In this systematic review, we aimed to characterize racial representation in genome-wide association studies (GWAS) of preterm birth. Our hypothesis was that, like in interventional clinical trials, non-Hispanic Black individuals would be underrepresented in GWAS studies despite their over-representation among preterm births.

## Review

Protocol and registration

This systematic review was conducted following the Preferred Reporting Items for Systematic Reviews and Meta-Analyses (PRISMA) guidelines (Supplemental Material 1) [12]. On June 11, 2020, a search of Scopus was performed. On June 12, 2020, searches of CINHAL, EMBASE, and MEDLINE (PubMed) were performed. We used syntax that targeted the main themes of genomics and preterm birth in humans (Supplemental Material 2). We registered this systematic review in the PROSPERO database (protocol # 380853). The registration is available in full through the National Institute for Health Research website [13]. All references identified during these searches were imported into the systematic review software Covidence (Veritas Health Innovation, Melbourne, Australia; available at www.covidence.org). Institutional review board approval was not required as identifiable data was not utilized in the course of this project.

Eligibility criteria and study selection

Eligible studies were primary publications reporting on genomic evaluation of the fetal or parental genome with length of gestation or preterm birth in humans and a sample size of at least 10 individuals. We included GWAS and whole exome or whole genome sequencing studies that examined at least 1,000 single nucleotide polymorphisms (SNPs) or genes. Studies were eligible if they were published in English with full text available. We excluded animal studies, meta-analyses or reviews that did not contain primary data, case reports, studies of single families, non-genomic studies (transcriptomics or methylomics), and studies that did not evaluate the outcome of preterm birth or length of gestation.

Two investigators (S.G. and R.L.) independently reviewed all abstracts and two authors (L.R. and S.G.) independently reviewed all full-text manuscripts. Studies were excluded if they did not contain any information regarding race, ethnicity, or ancestry of the participants in the primary manuscript text, tables, or supplementary data. Corresponding authors for each of the studies missing race, ethnicity, or ancestry were contacted in an attempt to acquire these data. Conflicts regarding abstract classification and full-text classification were resolved by a third author (H.H.B.). We also reviewed the reference lists of included manuscripts to identify additional relevant manuscripts meeting the inclusion criteria.

Data collection process

Race and ethnicity, as used in this systematic review, were categorized as they were reported in the included studies. Some studies reported on race and ethnicity separately. Other studies used mutually exclusive race and ethnicity categories, such as non-Hispanic Black. When individual studies used more specific terms, including Finnish, Danish, and Norwegian, we included these specific ethnicities, as described. Additionally, some studies reported on ancestry, rather than race or ethnicity. We also included ancestry groups, such as European, as defined by included studies and by DNA ancestry company 23andMe [14]. Details of each study, including technology type; sample type; year; location; total sample size (n); preterm n; racial, ethnic, or ancestral group; and number of genes or SNPs analyzed were extracted by two investigators (L.R. and S.G.). Discrepancies in extracted data were reviewed and resolved by a third author (R.L.).

Data analysis

The proportion of participants included in genomic studies of preterm birth represented by each race, ethnicity, or ancestry was the primary outcome for this systematic review. Participants within each study included infants and/or parents, as studies varied in from whom the genomic material was collected. We included a few studies that used data from overlapping datasets. Participants from overlapping datasets were only counted in our summed study sample size once, to prevent double counting the same participants. We then calculated the proportions of participants in each race, ethnicity, or ancestry category. All analyses were conducted using SAS 9.4 (SAS Institute Inc., Cary, NC).

Results

Study Selection

**Figure 1:**
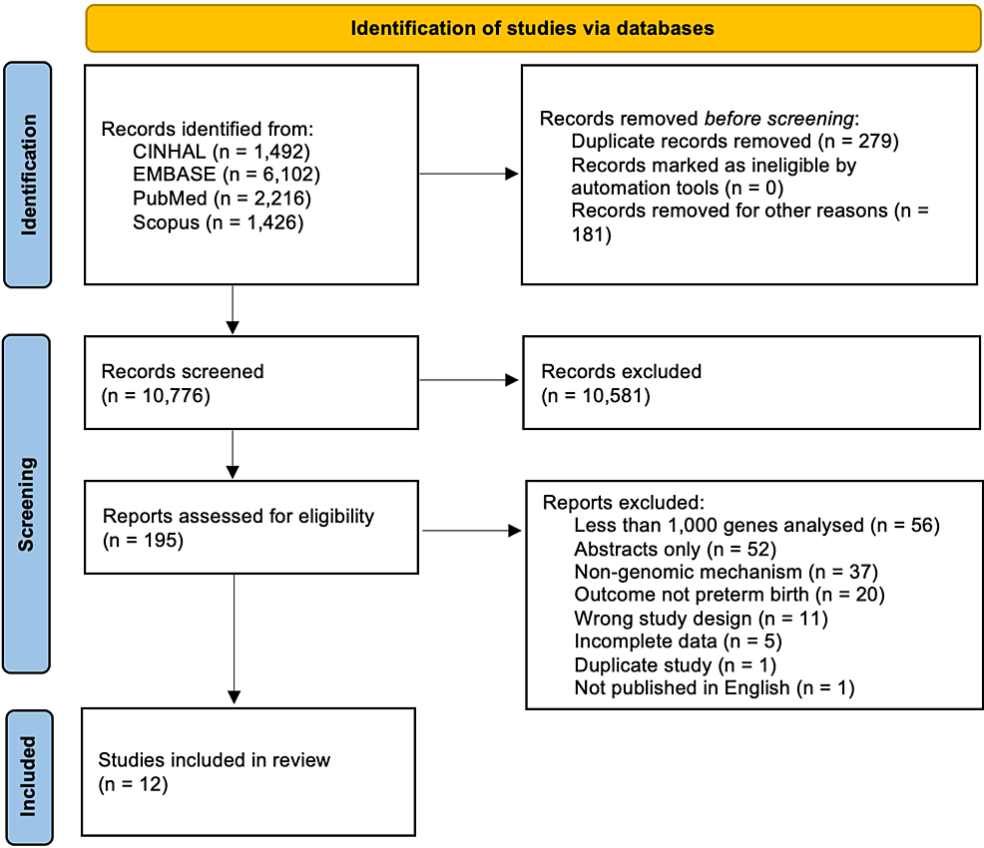
Study selection From: Page MJ, McKenzie JE, Bossuyt PM, Boutron I, Hoffmann TC, Mulrow CD, et al. The PRISMA 2020 statement: an updated guideline for reporting systematic reviews. BMJ 2021;372:n71. doi: 10.1136/bmj.n71 For more information, visit: http://www.prisma-statement.org/

Figure 1 describes the study selection process. There were 10,776 references identified from the search. Of the 195 abstracts that met initial inclusion criteria, full-text review led to the inclusion of 12 studies. Studies were excluded for the following reasons: 56 studies examined fewer than 1000 genes; 52 studies did not include full text, 37 studies did not include a genomic mechanism; 20 studies did not include preterm birth or gestational age as an outcome; and the remainder were the wrong study design.

Racial and Ethnic Representation Across All Studies

A total of 148,644 births (n=12,114 preterm births) were included from the 12 eligible studies. Table 1 describes the total breakdown of births by race, ethnicity, and ancestry reported. Either race, ethnicity, or ancestry was reported for each individual, with the exception of 880 individuals for whom both race and ethnicity were reported [15]. Ancestry was reported for 139,189 (93.6%) participants. Race was reported for 4,841 (3.3%) participants and ethnicity was reported for 7,154 (5.0%) participants.

**Table 1:** Breakdown of participants by race, ethnicity, or ancestry reported (n=148,644). *Percentages do not add to 100 as sometimes race and ethnicity were reported for the same study participants (n=880).

Patient Race, Ethnicity, or Ancestry	Participants (n=148,644)	Percent*
Race Alone		
White	1,853	1.25
Black or African American	2,444	1.64
Asian	96	0.07
Other	448	0.30
Ethnicity Alone		
Hispanic	590	0.40
Non-Hispanic	2,151	1.45
Danish	1,798	1.21
Norwegian	1,921	1.29
Danish & Norwegian	209	0.14
Finnish	666	0.45
Other	179	0.12
Ancestry (categories from 23andMe)		
Sub-Saharan African	1,917	1.29
East Asian & Native American	324	0.22
European	134,805	90.7
Western Asian & North African	0	0.00
Central & South Asian	0	0.00
Melanesian	0	0.00
Other	2,143	1.44

When classified by race alone, births among Black or African American individuals comprised the majority of births in this subset, with 2,444 births of 4,841 births (50.5%) [10, 11, 16-17]. Ethnicity was categorized into Hispanic, non-Hispanic, Danish, Norwegian, Danish and Norwegian, Finnish, and Another ethnicity [18-19]. The non-Hispanic group comprised 2,151 births (33.0%). Lastly, when classified by ancestry alone, European ancestry represented the vast majority of births (n=134,805, 90.7% of the total births included and 96.9% of births in the ancestry subset) [19-21].

Description of Racial, Ethnic, and/or Ancestral Representation in Individual Studies

Table 2 describes each eligible study included. The two largest cohorts among these 12 studies were from 1) Liu et al., a GWAS that included 80,970 participants (n=3,046 preterm births) from the Early Growth Genetics consortium, the initiative for Integrative Psychiatric Research, and the Genomic and Proteomic Network for Preterm Birth Research [22] and 2) Zhang et al., the 23andMe research cohort that included 43,568 participants (n=3,331 preterm births) [23]. Liu et al. conducted a GWAS meta-analysis to identify fetal genetic variants associated with gestational duration. This study only included individuals of European ancestry [22]. Similarly, the study by Zhang et al. restricted analyses to participants of >97% European ancestry [23]. These two studies were the largest cohorts and both were composed solely of patients with European ancestry. The next largest cohort was from the Health and Retirement Study, which was another GWAS [17]. In this cohort, 13,944 births were included, and 1,349 were preterm. This cohort was more heterogeneous, with 1,874 (13.4%) participants of sub-Saharan African ancestry, 249 (1.8%) of East Asian/Native American ancestry, 9,890 (70.9%) of European ancestry, and 1,847 (13.2%) classified as Another ancestry.

**Table 2:** Characteristics and findings of included genomics studies *: The total n in the discovery cohort of this study is n=84,689, however, this included data from two studies we had previously included in this cohort, so we removed the overlapping dataset n’s from this cohort. **: This study had an included n of 816, however, the n we received from the parent study authors with race and ethnicity data was n=880. PTB: preterm birth; SNP: single-nucleotide polymorphism; BMI: body mass index

Cohort and Setting	Year of Study	Publication Title Included in Systematic Review	First Author	Total n	Preterm n	Racial/ethnic/ancestral group(s) represented	How race/ ethnicity/ancestry was reported	Genes analyzed (n)	SNPs analyzed (n)	Findings	If non-homogenous study population: were disparities in race/ethnicity/ancestry and PTB reports in results?
Medical College of Virginia Hospitals, Richmond, VA & Hutzel Hospital, Detroit, MI	2017	Rare mutations and potentially damaging missense variants in genes encoding fibrillar collagens and proteins involved in their production are candidates for risk for preterm premature rupture of membranes	Bhavi Modi [10]	69	49	African American (n=69)	Self-reported race	188	Not specified	Rare heterozygous, nonsense, frameshift and damaging missense mutations were more prevalent in the genomes of neonates born of pregnancies complicated by PPROM compared to normal term controls.	Homogeneous study
Genomic and proteomic network for PTB research	2015	A genome-wide association study of early spontaneous preterm delivery	Heping Zhang [11]	2,040	1,025	Race: White (n=1,386), Black (n=459), Other (n=195) Ethnicity: Hispanic (n=416)	Self-reported race	Not specified	779,326	No maternal SNPs achieved a genome-wide significance level but several neonatal SNPs passed the genome-wide threshold. These results were not conclusive in a separate independent cohort.	No
Genomic and proteomic network for PTB research & Boston birth cohort	2017	Genome-wide approach identifies a novel gene-maternal pre-pregnancy BMI interaction on preterm birth	Xiumei Hong [16]	1,733	698	African American (n=1,733)	Race/ethnicity (race used in this study)	Not specified	2,160,368	A significant genome-wide interaction between maternal genotype rs11161721 in the COL24A1 gene and pre-pregnancy BMI on PTB in Black mothers from the Boston birth cohort and from an independent GWAS dataset	Homogeneous study
Genomic and proteomic network for PTB research, Early Growth Genetics Consortium, & Integrative Psychiatric Research	2019	Variants in the fetal genome near pro-inflammatory cytokine genes on 2q13 associate with gestational duration	Xueping Liu [22]	80,970*	3,046*	European ancestry (n=80,970)	Ancestry	Not specified	7,500,000	The rs7594852 SNP at the 2q13 locus was most significantly associated with gestational duration (postterm birth and not preterm or early preterm birth). Genetic variation at the locus was most strongly associated with timing of parturition in the later stages of pregnancy.	Homogeneous study
Oulu and Tampere University hospitals, health and retirement study cohort	2019	Risk of spontaneous preterm birth and fetal growth associates with fetal SLIT2	Heli Tiensuu [19]	666	247	European ancestry (n=666)	Self-report ethnicity	Not specified	6,778,521	SLIT2 variant and ROBO1 in placental cells are correlated with susceptibility to spontaneous preterm birth.	Homogeneous study
Health and retirement study cohort	2018	A genome-wide association study identifies only two ancestry-specific variants associated with spontaneous preterm birth	Nadav Rappoport [17]	13,944	1,349	Sub-Saharan African ancestry (n=1,874), East Asian/Native American ancestry (n=249), European ancestry (n=9,890), Other ancestry (n=1,847)	Race via birth certificate records	Not specified	2,015,750	Two loci associated with spontaneous PTB at a genome-wide level of significance (one variant in the African population and one in the American population)	No
Inova Fairfax Medical Center birth cohort	2019	Genomic and molecular characterization of preterm birth	Theo Knijnenburg [20]	791	270	Sub-Saharan African ancestry (n=43), East Asian/Native American ancestry (n=75), European ancestry (n=377), Ad-mixed American ancestry (n=127), Mixed ancestry (n=169)	Ancestry	10,773	Not specified	This study identified 72 candidate biomarker genes (involved in inflammation- and immunity-related pathways) for very early PTB.	No
Inova Translational Medicine Institute’s Molecular Study of Preterm Birth	2017	Fetal de novo mutations and preterm birth	Jingjing Li [15]	816	292	Race:** Asian (n=96), Black or African American (n=64), Native American (n=3), Other (n=94), Unknown (n=156), White or Caucasian (n=467) Ethnicity: Hispanic or Latino (174), not Hispanic or Latino (n=527), Unknown (n=179)	Self-reported questionnaires and EMR data extraction	Whole genome	Not specified	There is a significant association with PTB occurrence and de novo mutations in fetal genomes.	No
Danish National Birth Cohort	2013	A Genome-Wide Association Study of spontaneous preterm birth in a European population	Wilfred Wu [18]	1,798	849	European descent (n=1,798)	Self-reported ethnicity	Not specified	544,675	No genome-wide significant associations were found.	Homogeneous study
Norwegian Mother and Child Cohort	2016	Literature-Informed Analysis of a Genome-Wide Association Study of Gestational Age in Norwegian Women and Children Suggests Involvement of Inflammatory Pathways	Jonas Bacelis [21]	1,921	882	Norwegian (n=1,921)	Self-reported ethnicity	Not specified	525,577	No genome-wide significant associations with gestational age were found. Thirty-two genes were highlighted for future research as having possible contribution to gestational age in deliveries that start with labor.	Homogeneous study
23andMe research cohort	2017	Genetic Associations with Gestational Duration and Spontaneous Preterm Birth	Ge Zhang [23]	43,568	3,331	European ancestry (n=43,568)	Ancestry via DNA extraction and genotyping	Not specified	15,635,593	Variants at multiple loci (EBF1, EEFSEC, AGTR2, WNT4, ADCY5, and RAP2C) were associated with gestational duration and variants at the EBF1, EEFSEC, AGTR2 loci were associated with preterm birth.	Homogeneous study
Medical College of Virginia Hospitals, Richmond, VA & Hutzel Hospital, Detroit, MI	2018	Discovery of rare ancestry-specific variants in the fetal genome that confer risk of preterm premature rupture of membranes (PPROM) and preterm birth	Bhavi Modi [10]	119	76	African American (n=119)	Self-reported race	20,000	Not specified	Multiple rare population-specific variants in the fetal genome (DEFB1 and MBL2) contribute to preterm birth associated with PPROM	Homogeneous study

Discussion

Of the 148,644 births in this systematic review of GWAS of preterm birth, over 80% of births were from two studies and over 90% of those study participants were of European ancestry from two large cohort studies [22,23]. While studies that used race and ethnicity designations had more diversity, their sample sizes were much smaller, totaling 24,106 births.

Reporting of race, ethnicity, and ancestry is not uniform in GWAS of preterm birth. Thus, our ability to extract information on race and ethnicity into pre-specified categories was limited, in part, by lack of consensus guidelines on how race and ethnicity should be reported [24]. To our knowledge, this is the first systematic review to specifically evaluate the classification of patients in the genomics of preterm birth. However, in a systematic review evaluating race and ethnicity representation in DNA methylomic studies of preterm birth, we found that Black and non-Hispanic Black individuals represented 28% of the participants among 16 relevant studies and thus were overall well-represented. Some reporting bias may have been present due to the lack of race and ethnicity data published in large studies from European countries [25]. Granular information from these larger studies may help to illuminate inequities in representation within those studied populations.

The lack of individuals with a substantial proportion of African ancestral genetic sequences in the largest two GWAS analyses of preterm birth to date is problematic due to the overwhelming burden of preterm birth among Black people (who also are likely to have meaningful proportions of African ancestral genes) in the US. However, it is possible that some individuals in the studies identified as Black even if they had >97% European ancestry. Recently, a consortium of The National Heart, Lung and Blood Institute, Trans-Omics for Precision Medicine, developed recommendations on the reporting of race, ethnicity, and ancestry in genetic research [26]. These include distinguishing between non-genetic reported information and genetically inferred information and using bias-free language regarding racial and ethnic identity. Recognizing the challenge in combining data from multiple studies with variability in data collection methods and categories, the consortium also recommended clearly describing source data for race and ethnicity information and preserving specific population information rather than prematurely collapsing distinct populations into broader categories.

Data from studies that use ancestral designations are insufficient to claim that racial disparities in preterm birth are genetic in origin for three reasons. First, there is not a specific high-risk genotype that predicts preterm birth identified from existing studies. The study by Zhang et al. found associations with the length of gestation, but no strong signal with respect to any gene and preterm birth. Wu et al. did not find a significant genetic association with spontaneous preterm birth in a large European population studied [18]. Modi et al. identified two rare variants of African ancestry associated with preterm premature rupture of membranes, but not directly associated with preterm birth. Second, the largest GWAS analyses of preterm birth have not included enough genetic diversity to quantify the extent to which genetics might explain differences in preterm birth rates by ancestry [27]. Third, ancestry, while it can be somewhat correlated with race, is not the same as race [28]. Race and ancestry are distinct concepts. Race is defined as a non-biological social construct and is often used interchangeably with ethnicity. In contrast, ancestry refers to one’s biological ancestors from whom DNA is inherited and thus implies an individual’s genetic origins [26]. There are diseases that vary in their prevalence across racial groups due to single gene mutations, such as sickle cell disease and cystic fibrosis [29, 30]. However, for complex, multifactorial disorders, such as hypertension and preterm birth, social and environmental exposures that differ across racial designations play a large role in inequities. Third, the majority of studies included in this systematic review included homogeneous populations only. Rappoport et al. included multiple races in the study cohort and acknowledged the limitation of race categorization given the complex interaction between genotypes and phenotypes [17]. Other studies that included multiple races or ancestries failed to comment further on how their findings helped to explain inequities in preterm birth.

When applied to race, ethnicity, ancestry and preterm birth, genomics has the danger of further marginalizing groups, and it is critical that studies on genomics and preterm birth are well-balanced and have appropriate representation of individuals with African ancestry and who identify as Black.

One strength of this study is our inclusion of GWAS. We excluded candidate gene studies, which are studies that focus on associations between genetic variation within pre-specified genes of interest. Some may argue that candidate gene studies may have some inherent racial bias by including/excluding certain groups among their populations of study. Instead, by examining broader GWAS, we hoped to decrease selection bias.

Limitations of this study include that, as described above, the reporting of race, ethnicity, and ancestry is not uniform, which makes comparison among studies challenging. Additionally, there is some heterogeneity in terms of sample type (fetal versus parental genome) among studies, which makes comparison impossible. Finally, given that we used broad search terms to avoid missing any relevant studies, there was a large number of abstracts to review. This took a considerable amount of time; thus, we did not include manuscripts that may have been published in the last nearly three years.

## Conclusions

We found that the largest GWAS analyses of preterm birth included almost exclusively individuals of European ancestry. Studies that used race and ethnicity designations had more diversity but included fewer participants. This review highlights the importance of accurate terminology and description in genomic studies of preterm birth. Despite the disproportionate impact of preterm birth on Black individuals and also individuals of African ancestry, these populations are under-represented in genomic research on preterm birth. The inclusion of these populations and accurate description of race, ethnicity, and ancestry are paramount to robust and meaningful research of genomics and preterm birth.

## Appendices

Supplemental material 1

**Table 3:** PRISMA 2020 checklist From:  Page MJ, McKenzie JE, Bossuyt PM, Boutron I, Hoffmann TC, Mulrow CD, et al. The PRISMA 2020 statement: an updated guideline for reporting systematic reviews. BMJ 2021;372:n71. doi: 10.1136/bmj.n71 For more information, visit: http://www.prisma-statement.org/

Section and Topic	Item #	Checklist item	Location where item is reported
TITLE			
Title	1	Identify the report as a systematic review.	Title page
ABSTRACT			
Abstract	2	See the PRISMA 2020 for Abstracts checklist.	Manuscript
INTRODUCTION			
Rationale	3	Describe the rationale for the review in the context of existing knowledge.	Manuscript
Objectives	4	Provide an explicit statement of the objective(s) or question(s) the review addresses.	Manuscript
METHODS			
Eligibility criteria	5	Specify the inclusion and exclusion criteria for the review and how studies were grouped for the syntheses.	Manuscript, Figure 1
Information sources	6	Specify all databases, registers, websites, organisations, reference lists and other sources searched or consulted to identify studies. Specify the date when each source was last searched or consulted.	Manuscript, supplemental Table 1
Search strategy	7	Present the full search strategies for all databases, registers and websites, including any filters and limits used.	Manuscript, supplemental Table 1
Selection process	8	Specify the methods used to decide whether a study met the inclusion criteria of the review, including how many reviewers screened each record and each report retrieved, whether they worked independently, and if applicable, details of automation tools used in the process.	Manuscript, Figure 1
Data collection process	9	Specify the methods used to collect data from reports, including how many reviewers collected data from each report, whether they worked independently, any processes for obtaining or confirming data from study investigators, and if applicable, details of automation tools used in the process.	Manuscript, supplemental Table 1
Data items	10a	List and define all outcomes for which data were sought. Specify whether all results that were compatible with each outcome domain in each study were sought (e.g., for all measures, time points, analyses), and if not, the methods used to decide which results to collect.	Manuscript
	10b	List and define all other variables for which data were sought (e.g., participant and intervention characteristics, funding sources). Describe any assumptions made about any missing or unclear information.	Manuscript, supplemental Table 1
Study risk of bias assessment	11	Specify the methods used to assess the risk of bias in the included studies, including details of the tool(s) used, how many reviewers assessed each study and whether they worked independently, and if applicable, details of automation tools used in the process.	Manuscript
Effect measures	12	Specify for each outcome the effect measure(s) (e.g., risk ratio, mean difference) used in the synthesis or presentation of results.	Manuscript
Synthesis methods	13a	Describe the processes used to decide which studies were eligible for each synthesis (e.g., tabulating the study intervention characteristics and comparing against the planned groups for each synthesis (item #5)).	Manuscript
	13b	Describe any methods required to prepare the data for presentation or synthesis, such as handling of missing summary statistics, or data conversions.	Manuscript
	13c	Describe any methods used to tabulate or visually display results of individual studies and syntheses.	Manuscript
	13d	Describe any methods used to synthesize results and provide a rationale for the choice(s). If meta-analysis was performed, describe the model(s), method(s) to identify the presence and extent of statistical heterogeneity, and software package(s) used.	Manuscript
	13e	Describe any methods used to explore possible causes of heterogeneity among study results (e.g., subgroup analysis, meta-regression).	Manuscript
	13f	Describe any sensitivity analyses conducted to assess the robustness of the synthesized results.	Manuscript
Reporting bias assessment	14	Describe any methods used to assess the risk of bias due to missing results in a synthesis (arising from reporting biases).	Manuscript
Certainty assessment	15	Describe any methods used to assess certainty (or confidence) in the body of evidence for an outcome.	Manuscript
RESULTS			
Study selection	16a	Describe the results of the search and selection process, from the number of records identified in the search to the number of studies included in the review, ideally using a flow diagram.	Manuscript, Figure 1
	16b	Cite studies that might appear to meet the inclusion criteria, but which were excluded, and explain why they were excluded.	Manuscript
Study characteristics	17	Cite each included study and present its characteristics.	Manuscript, Table 2
Risk of bias in studies	18	Present assessments of risk of bias for each included study.	Manuscript
Results of individual studies	19	For all outcomes, present, for each study: (a) summary statistics for each group (where appropriate) and (b) an effect estimate and its precision (e.g., confidence/credible interval), ideally using structured tables or plots.	Manuscript
Results of syntheses	20a	For each synthesis, briefly summarise the characteristics and risk of bias among contributing studies.	Manuscript
	20b	Present results of all statistical syntheses conducted. If meta-analysis was done, present for each the summary estimate and its precision (e.g., confidence/credible interval) and measures of statistical heterogeneity. If comparing groups, describe the direction of the effect.	Manuscript
	20c	Present results of all investigations of possible causes of heterogeneity among study results.	Manuscript
	20d	Present results of all sensitivity analyses conducted to assess the robustness of the synthesized results.	Manuscript
Reporting biases	21	Present assessments of risk of bias due to missing results (arising from reporting biases) for each synthesis assessed.	Manuscript
Certainty of evidence	22	Present assessments of certainty (or confidence) in the body of evidence for each outcome assessed.	Manuscript
DISCUSSION			
Discussion	23a	Provide a general interpretation of the results in the context of other evidence.	Manuscript
	23b	Discuss any limitations of the evidence included in the review.	Manuscript
	23c	Discuss any limitations of the review processes used.	Manuscript
	23d	Discuss implications of the results for practice, policy, and future research.	Manuscript
OTHER INFORMATION			
Registration and protocol	24a	Provide registration information for the review, including register name and registration number, or state that the review was not registered.	Title page, References
	24b	Indicate where the review protocol can be accessed, or state that a protocol was not prepared.	Manuscript
	24c	Describe and explain any amendments to information provided at registration or in the protocol.	Not relevant
Support	25	Describe sources of financial or non-financial support for the review, and the role of the funders or sponsors in the review.	Title page
Competing interests	26	Declare any competing interests of review authors.	Title page, conflict of interest forms
Availability of data, code and other materials	27	Report which of the following are publicly available and where they can be found: template data collection forms; data extracted from included studies; data used for all analyses; analytic code; any other materials used in the review.	Supplemental Table 1

Supplemental material 2

**Table 4:** Search terms and database records

Source	Search terms	Limits	# records
CINHAL	( ( ( (MH "Infant, Premature") OR (MH "Childbirth, Premature") or “Premature” OR “preterm birth” OR “PTB” ) AND ( (MH "Genetics") OR (MH "Gene Pool") OR (MH "Genetic Profile") OR (MH "Genetic Research") OR (MH "Genome Wide Association Study") OR (MH "Genomics") OR (MH "Genes") OR (MH "Genome") OR (MH "Gene Expression") OR (MH "Polymorphism, Genetic") OR (MH "Genotype") OR “GWAS” OR “Gene” OR “SNP” OR “Single nucleotide polymorphism” OR “Genetic” OR “genotype profile” OR genes OR genetic OR genetics OR genome ) ) AND ( (MH "Pregnancy") OR (MH "Childbirth") OR (MH "Labor") OR (MH "Pregnancy, High Risk") OR (MH "Expectant Mothers") OR (MH "Gestational Age") OR “Pregnant” OR pregnancy OR “Birth” OR “Gestation” OR “Pregnancies” OR “Childbirth” ) ) NOT ( ( (MH "Animals+") OR “animalia” OR “metazoa” OR animal OR animals ) NOT ( ( (MH "Animals+") OR “animalia” OR “metazoa” OR animal OR animals ) AND ( (MH "Human") OR “Homo Sapiens” OR human OR man ) ) )	January 1, 2000-June12, 2020; English language only	1,492
EMBASE	('immature and premature labor'/exp OR 'immaturity'/exp OR 'premature labor'/exp OR 'prematurity'/exp OR 'premature':ti,ab OR 'prematurity':ti,ab OR 'preterm':ti,ab) AND ('genome-wide association study'/exp OR 'genetic association study'/exp OR 'single nucleotide polymorphism'/exp OR 'snp'/exp OR 'genetic polymorphism'/exp OR 'genetic variability'/exp OR 'genotype'/exp OR 'gene'/de OR 'comparative genomics'/exp OR 'functional genomics'/exp OR 'structural genomics'/exp OR 'genetic profile'/exp OR 'genomic':ti,ab OR 'genomics':ti,ab OR 'genome':ti,ab OR 'genetic':ti,ab OR 'genetics':ti,ab OR 'gene':ti,ab OR 'genotype':ti,ab) AND ('pregnancy'/exp OR 'gestational age'/exp OR 'fetus'/exp OR 'pregnant woman'/exp OR 'pregnan*':ti,ab OR 'gravidity':ti,ab OR 'gestation*':ti,ab OR 'child bearing':ti,ab OR 'childbearing':ti,ab) NOT (('animal'/exp OR 'animal') NOT (('animal'/exp OR 'animal') AND 'human'/exp OR 'normal human'/exp OR 'human' OR 'man'))	January 1, 2000-June12, 2020; English language only	6,102
MEDLINE (PubMed)	((("Premature Birth"[Mesh] OR Premature Births [tiab] OR Premature Birth [tiab] OR Preterm birth [tiab] OR Preterm Births [tiab] OR "Infant, Premature"[Mesh:NoExp] OR premature infant [tiab] OR premature infants [tiab]) AND ("Genomics"[Mesh] OR “Genetics"[Mesh] OR genes [Mesh] OR genome-wide association study [Mesh] OR Single Nucleotide Polymorphism [MeSH] OR genetic variation [MeSH] OR genotype [MeSH] OR gene frequency [MeSH] OR genetic profile [MeSH] OR Genomics [tiab] OR Genetics [tiab] OR Genomic [tiab] OR Genetic [tiab] OR gene [tiab] OR genes [tiab] OR “Single Nucleotide Polymorphisms” [tiab] OR “Single Nucleotide Polymorphism” [tiab] OR SNPs [tiab] OR SNP [tiab] OR GWAS [tiab] OR genotype profile [tiab] OR genotype profiles [tiab]) AND ("pregnancy"[MeSH Terms] OR "pregnancy"[tiab] OR "pregnancies"[tiab] OR "pregnancy s"[tiab] OR pregnant [tiab] OR gestation OR “gestational age” [Mesh] OR childbearing [tiab] OR child bearing [tiab] OR "Fetus"[Mesh] OR fetus [tiab] OR fetal [tiab] OR "perinatal"[tiab] OR "perinatally"[tiab] OR "perinatals"[tiab] OR maternal [tiab])) NOT ((("Animals"[Mesh] or animal [tiab] or Animalia [tiab] or Animal [tiab] or Metazoa)) NOT ((("Animals"[Mesh] or animal [tiab] or Animalia [tiab] or Animal [tiab] or Metazoa)) AND (Humans[Mesh] OR Homo sapiens [tiab] OR Man [tiab] OR Human)))	January 1, 2000-June12, 2020; English language only	2,216
Scopus	((TITLE-ABS-KEY(premature OR prematurity OR immaturity OR preterm)) AND ((TITLE-ABS-KEY(gene OR genetic OR genetics) OR TITLE-ABS-KEY(genomic OR genomics OR genome)OR TITLE-ABS-KEY(genotype OR "Single Nucleotide Polymorphism" OR SNP OR GWAS))) AND ((TITLE-ABS-KEY(pregnan*) OR TITLE-ABS-KEY(gestation OR gestational) OR TITLE-ABS-KEY(gravidity) OR TITLE-ABS-KEY("child bearing" OR childbearing)))) AND NOT ((TITLE-ABS-KEY(animal OR animals)) AND NOT ((TITLE-ABS-KEY(animal OR animals)) AND (TITLE-ABS-KEY(human OR humans OR man )))) AND ( LIMIT-TO ( LANGUAGE,"English" ) ) AND ( LIMIT-TO ( PUBYEAR,2020) OR LIMIT-TO ( PUBYEAR,2019) OR LIMIT-TO ( PUBYEAR,2018) OR LIMIT-TO ( PUBYEAR,2017) OR LIMIT-TO ( PUBYEAR,2016) OR LIMIT-TO ( PUBYEAR,2015) OR LIMIT-TO ( PUBYEAR,2014) OR LIMIT-TO ( PUBYEAR,2013) OR LIMIT-TO ( PUBYEAR,2012) OR LIMIT-TO ( PUBYEAR,2011) OR LIMIT-TO ( PUBYEAR,2010) OR LIMIT-TO ( PUBYEAR,2009) OR LIMIT-TO ( PUBYEAR,2008) OR LIMIT-TO ( PUBYEAR,2007) OR LIMIT-TO ( PUBYEAR,2006) OR LIMIT-TO ( PUBYEAR,2005) OR LIMIT-TO ( PUBYEAR,2004) OR LIMIT-TO ( PUBYEAR,2003) OR LIMIT-TO ( PUBYEAR,2002) OR LIMIT-TO ( PUBYEAR,2001) OR LIMIT-TO ( PUBYEAR,2000) )	January 1, 2000- June 11, 2020; English language only	7,050
